# Clinician perspectives on designing and implementing a hereditary cancer transition clinic

**DOI:** 10.1186/s13053-024-00304-5

**Published:** 2025-01-11

**Authors:** Jazmine L. Gabriel, Victoria Schlieder, Jessica M. Goehringer, Tracey Leitzel, Emily Ann Sugrue, Sarah Zultevicz, Thomas W. Davis, Gemme Campbell-Salome, Katrina Romagnoli

**Affiliations:** 1 Department of Population Health Sciences, Geisinger, Danville, PA 17822 USA; 2 Department of Genomic Health, Geisinger, Danville, PA USA; 3 Geisinger Health System, Danville, PA USA; 4 Augustana University, Sioux Falls, SD USA

**Keywords:** Health care transition, Adolescent/young adult, Genetics/genomic medicine, Hereditary cancer predisposition, Contextual inquiry, Implementation science, Human-centered design

## Abstract

Early identification of hereditary cancer predisposition in adolescents and young adults represents a unique opportunity to target cancer prevention and improve survival in a population at risk for adverse health outcomes. However, adolescents and young adults face challenges unique to their stage of life that can undermine their transition from pediatric to adult healthcare and lead to interruptions in preventative care. The purpose of this study was to understand expert perspectives on factors relevant to designing and implementing a transition clinic for adolescents and young adults with hereditary cancer predisposition. We used qualitative methods informed by human-centered design and implementation science to identify implementation considerations rooted in clinician experience. To understand clinic design and clinician experience at Geisinger transition clinics, we conducted a contextual inquiry using clinic observations and follow-up interviews of clinicians. To learn about designing and implementing a transition program, we also conducted in-depth interviews with national transition experts actively involved in developing, implementing, or participating in transition clinics around the United States. The contextual inquiry resulted in three diagrams depicting the following common elements of transition clinics at our institution: relationship building with patients, care coordination, stepwise transition education, communication between providers, and a sustainable clinic home. Interviews were analyzed deductively using thematic analysis to learn clinician perspectives about program implementation specific to each domain of the RE-AIM theoretical framework: reach, effectiveness, adoption, implementation, and maintenance.

## Background

More than two million new cancer cases are projected for 2024, and eighty-four thousand will be diagnosed in adolescents and young adults (AYA) ages 15–39 [22, 61]. Approximately 5–10% of cancers, particularly cancers diagnosed before age 50, are caused by a germline pathogenic or likely pathogenic (P/LP) variant in a cancer predisposition gene [31, 67]. Individuals identified to have a germline P/LP variant through pre-symptomatic genetic testing can significantly reduce their risk of cancer by adhering to condition-specific recommendations for surveillance, chemoprevention, and prophylactic surgery [31, 62]. Early identification of cancer predisposition in AYA represents a unique opportunity to target cancer prevention and improve survival in a population at risk for adverse health outcomes. However, AYA face challenges unique to their stage of life that can undermine their transition from pediatric to adult healthcare and lead to interruptions in preventative care [6, 39].

To support AYA and reduce care gaps as they transition from child/family-centered health care to adult health care, the American Academy of Pediatrics (AAP), American Academy of Family Physicians (AAFP), and American College of Physicians (ACP) created a joint clinical report to systematize healthcare transition (HCT) and show how the elements of transition can be adapted to different models of care [3, 65]. Increasingly, medical practices, health systems, and public health programs are designing and implementing HCT programs for AYA with chronic health conditions [68]. While some multi-system genetic conditions with increased risk for cancer (e.g., tuberous sclerosis) feature prominently in the transition literature, transition care for individuals with hereditary cancer predisposition syndromes remains underexplored [2, 25, 52]. In addition, few studies address clinician experiences with designing, implementing, and sustaining HCT programs for individuals at increased risk for cancer [25, 48, 65].

A general discussion of implementation approaches for new HCT programs is included in the updated clinical report on HCT from the AAP, AAFP, and ACP [65]. However, there is no discussion of implementation considerations informed by theoretical frameworks from implementation science. Implementation science, the study of methods to promote uptake of research findings and evidence-based practices, can help identify and address barriers and facilitators to HCT implementation [7, 24]. The RE-AIM framework is a validated implementation science framework to guide intervention development and evaluation by specifying domains important for successful adoption and implementation of effective interventions. While RE-AIM was initially developed to guide program evaluation, it has also been used to plan programs [9, 33, 44]. Attention to the following domains in all phases of research can improve the odds of public health impact: reaching the target population (R), effectiveness of the intervention (E), adoption of the intervention by settings and individuals (A), implementation and fidelity to the intervention protocol (I), and maintenance of the intervention six months after implementation (M) [9, 29, 32, 42]. We selected the RE-AIM framework in order to approach clinic design with outcomes in mind.

Developing intervention ideas in collaboration with the intended audience can improve the likelihood of successful interventions [34]. Perspectives and experiences of organizational leaders, patients, and clinicians all warrant in-depth consideration. As a first step, this study combines implementation science and human centered design (HCD) to engage clinician stakeholders as partners in identifying factors for designing and implementing a transition clinic for AYA with hereditary cancer predisposition.

HCD (also called user-centered design and design thinking) uses multi-disciplinary methods to focus on people and their context to ensure the correct problems are identified prior to designing innovative solutions [1, 37, 40]. This creative, iterative approach to problem-solving begins by empathizing with human needs and experiences to design solutions that are desirable, feasible, and viable [43]. As a pre-implementation study, we began with the *inspiration* phase of HCD, which involves empathizing with users and understanding the current state. HCD also includes an *ideation* phase, which involves brainstorming, designing, and iteratively refining prototypes, as well as an implementation phase. The goal of the inspiration phase is not to arrive at a solution but to ground these future stages in an in-depth understanding of human needs and their everyday context. [18].

Combining implementation science with HCD can increase the likelihood of successfully implementing innovative programs like HCT by understanding the experience and context of program participants and specifying the implications of their experiences along several key domains relevant to implementation [18, 23, 38, 42, 46]. Implementation science offers theories, models, and frameworks for examining multi-level determinants, while HCD offers in-depth understanding of everyday user-experiences and context that increase the odds of intervention fit. Like implementation science, HCD frequently involves considering individuals beyond primary users i.e., not only patients but also clinic leaders who implement interventions and family members of patients [18]. We focused first on the clinician perspective of designing and implementing a transition program, but future work will identify patient and parent needs and clarify system-level priorities.

### Objective

This project is part of a pre-implementation study to inform development of a transition clinic for AYA with hereditary cancer predisposition. Our objective was to understand expert perspectives on factors relevant for designing and implementing a transition clinic for this population. Using qualitative methods informed by HCD and implementation science, we addressed the following research questions (RQs):

1. What can we learn from clinician experiences with designing, implementing, and operating a transition clinic to inform development of a future transition clinic for AYA with hereditary cancer predisposition?
2. What factors are relevant to designing and implementing a transition clinic for AYA with a hereditary cancer predisposition syndrome?

## Methods

### Study setting

Geisinger is an integrated healthcare system that serves a largely rural, geographically stable population in Pennsylvania and has precision health research initiatives consisting of whole exome sequencing results for pediatric and adult patients for over 35 actionable genetic conditions [17, 58, 60]. While transition clinics exist at Geisinger, no transition program exists for individuals with hereditary cancer predisposition.

### Study design and procedures

This study was reviewed and approved by both the Geisinger Institutional Review Board (IRB 2021–0764) and the Pennsylvania Department of Health Institutional Review Board. We used multiple methods informed by HCD and implementation science to identify factors relevant for implementing a transition program for AYA with hereditary cancer. To understand the current state of transition programs at our institution, we conducted a contextual inquiry, and to gain inspiration for innovative program design, we focused on expert experiences with analogous models of care around the country.

We conducted a contextual inquiry using clinic observations and follow-up interviews of clinicians to understand clinic design and clinician experience at Geisinger transition clinics. Participants were eligible for participation in the contextual inquiry if they were currently working at a transition clinic at Geisinger. They were identified in consultation with a transition expert (TD) on the study team. Contextual inquiry, a method of HCD, is a field research method in which data collection occurs in the person’s work and life context to gain a thorough understanding of the day-to day environment in which a given intervention will be used [11, 66]. Interviews frequently follow observations so that the researcher can inquire into specifics observed and gain additional insights. The aim is to ensure fit between the person’s needs and the developed solution by understanding what people are doing and why. Contextual inquiry was done by two experienced qualitative researchers (JG and VS) trained by a study team member (KR) with expertise in HCD. Observations and follow-up interviews were completed between May and October of 2023.

To learn about barriers and facilitators to implementing a transition program, we also conducted in-depth interviews via phone or videoconference with national transition experts actively involved in developing, implementing, and/or participating in transition clinics around the United States. We used purposive and snowball sampling to identify national transition experts working at clinics outside of Geisinger. Participants were eligible for an interview if they worked clinically at a transition clinic in the United States. Interview topics were informed by RE-AIM constructs and the Six Core Elements of HCT™ described by the organization Got Transition^®^ and recommended by the AAP, AAFP, and ACP [42, 65].

Topics included experience developing and implementing a transition clinic, factors contributing to organizational adoption and patient attendance, approaches to preparing for transition of AYA to adult health care, and considerations for clinic sustainability over time (Table S1). All interviews occurred between July and November of 2023. Participants completed a brief demographic survey in addition to their interview and received a $50 gift card for their participation. Interviews were audio-recorded and transcribed verbatim by a professional transcriptionist. Following the interview, interviewers summarized key content using a matrix designed to facilitate rapid analysis of RE-AIM domains. [8, 54].

### Data analysis

Data collected from the contextual inquiry (clinic observations and follow-up interviews with clinicians) were analyzed by creating visual diagrams of qualitative data to depict essential aspects of clinic design and clinician experience [12, 45, 55]. Diagrams were created in PowerPoint using an iterative approach in which researchers met biweekly to discuss which components of diagrams best captured essential aspects of each clinic. To ensure that diagrams accurately captured clinic elements, clinicians from each clinic were engaged as co-creators of the diagram: interviewees from each clinic reviewed a copy of the diagram and participated in a team meeting to discuss and further refine it.

Interviews with national experts were analyzed using a rapid assessment procedure (RAP), an iterative, team-based approach to qualitative analysis that uses a summary template or matrix to increase analytical efficiency without compromising rigor [8, 54]. Following each interview, research team members (JG, VS) entered data into a matrix of domains corresponding to interview questions. An iterative cycle of data collection and analysis followed. After eight interviews, we used the rapid analysis matrix to assess whether the depth, diversity, and complexity of the data were sufficient to address our research questions in a meaningful way. We identified rich data addressing all five RE-AIM constructs. We then completed a ninth interview with an expert whose experience using multiple models of transition care contributed additional depth to our analysis.

After the rapid analysis was complete, the first author (JG) open-coded the transcripts in Atlas.ti (qualitative data analysis software) to increase familiarity with the content not captured by the summary template and to ensure that important aspects of the data would not be missed in further phases of analysis [30]. Data was then deductively analyzed by RE-AIM construct, using definitions from Holtrop et al. [42]. Initial themes were developed using thematic analysis and were refined through iterative discussion with four team members (JG, VS, GCS, KR), including two senior researchers with expertise in qualitative methods and implementation science [15].

Due to questions raised about the concepts of data saturation, thematic saturation, and information redundancy in qualitative research, including whether it is possible to find ‘nothing new’ as long as new interviews and interpretive analysis continue, the team focused on the quality of the data, rather than the apparent absence of novel content, to determine *theoretical sufficiency* [14–16, 21, 27, 41, 47, 50, 56, 57, 64]. Following Braun & Clarke, we assume that meaning is context-dependent and requires interpretation. Accordingly, the decision to stop interviewing and proceed with each stage of analysis requires an interpretative judgment related to the purpose of the study, and it is not always possible to locate the precise number of transcripts at which no new interpretations are possible [16].

## Results

### Findings on RQ1 from contextual inquiry

**Fig. 1:**
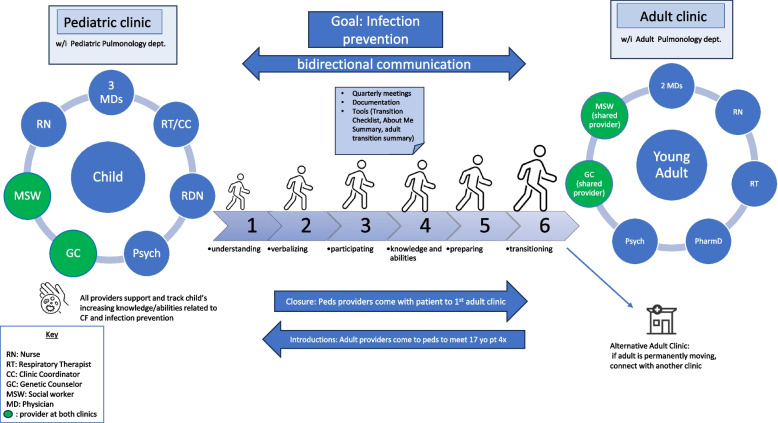
Cystic fibrosis transition clinic: key elements

**Fig. 2:**
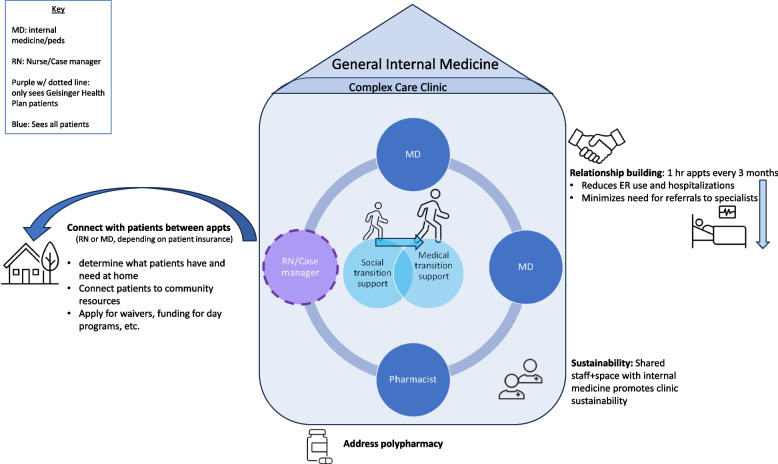
Complex care clinic: key elements

**Fig. 3:**
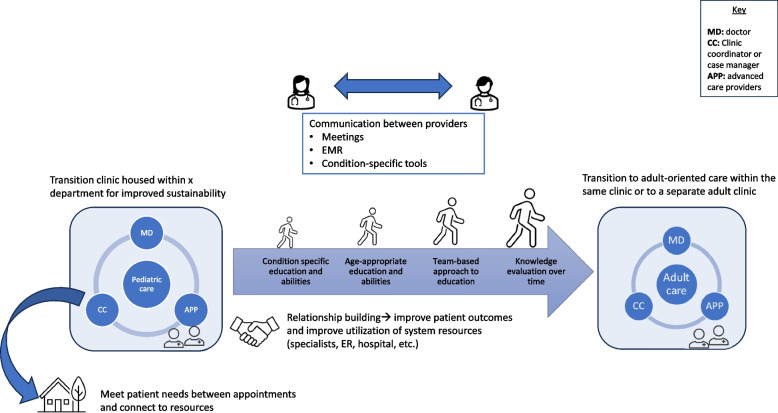
Generic transition clinic: key elements of both clinics

To address RQ1, we completed 19 hours of contextual inquiry at two Geisinger-based transition clinics. A total of 15 clinicians and 16 patients were observed during clinic observations at Geisinger. One clinic was a pediatric cystic fibrosis clinic that provides care and transition preparation for individuals 0–18 years old prior to their transfer to the adult cystic fibrosis clinic. The other was a person-centered, complex-care clinic within the department of General Internal Medicine, providing care for patients of all ages with complex health and social needs due to multiple chronic conditions. Results from the contextual inquiry include descriptions of each clinic’s approach to transition care, two diagrams that map essential elements of each clinic, and one diagram abstracting from each clinic diagram to depict key elements of a successful transition clinic at our institution (Figs. 1, 2 and 3). Common elements included relationship building with patients, care coordination, stepwise transition education for patients and parents, communication between providers, and a sustainable clinic home. These interrelated dimensions are described for each clinic below.

#### Cystic fibrosis clinic

For AYA with cystic fibrosis, transition to adult care was facilitated by way of a collaboration between the multidisciplinary pediatric and adult cystic fibrosis clinics (Fig. 1). Quarterly pediatric appointments are one to three hours long and characterized by regular review of condition-specific information and abilities, tailored to patient age and maturity. Careful documentation of patients’ condition-specific knowledge and abilities, regular communication between the clinics, and a warm-hand-off help to ensure a smooth transition from pediatric to adult care. The care-coordinator, along with nurses, address patient needs between appointments.

Transition preparation is divided into six stages. Beginning at age 10, patients are educated about their condition and the transition process. Knowledge expectations increase and parental involvement decreases as the child progresses through the six stages. Each clinician takes responsibility for ensuring that knowledge advances in a stepwise, age-appropriate manner. A checklist is used to track the child’s progress in different domains, with each team member indicating whether a given item (e.g., understands good handwashing and avoiding germs) has been discussed and whether the child’s understanding is “in progress” or “proficient.” Progress in each area is then recorded in the electronic medical record (EMR), where all team members can review it, identify needs between appointments, and plan goals for next appointments.

When the adolescent reaches stage six, generally at age 17, the adult team members come to the pediatric side to meet the patient and family. They evaluate transition readiness and accompany the patient to the adult clinic. Prior to completing the transition to adult care, the pediatric clinicians will send transfer documents to the adult team to communicate patient priorities: one document, called “About Me,” is completed by the patient and specifies patient preferences and concerns; the other document, called, “Adult Transition Summary Sheet” includes patient-specific medical information, such as medical history and current medical plan.‬‬‬‬‬‬‬‬‬‬‬‬‬‬‬‬‬‬‬‬‬‬‬

#### Complex care clinic

For individuals with complex care needs, transition to adult care occurs within one clinic that serves patients of all ages. Clinicians involved and other key dimensions of the clinic are shown in Fig. 2. Transition is tailored to the patient’s needs and abilities, with some patients taking on more responsibilities for their own care than others. The approach is characterized by relationship building within hour long appointments and care coordination to meet patient needs between appointments.

The complex care clinic serves patients with different medical conditions and social and behavioral needs that require varying degrees of support. Thus, there is no uniform approach to patient education, as in the cystic fibrosis clinic. Some patients will never fully take responsibility for their own care due to their underlying medical condition. However, there are certain aspects of transitioning to adult care that happen regardless of a patient’s abilities (e.g., changing insurance when parental insurance is no longer an option). Other transitions are more specific to the patient’s abilities, including issues of guardianship, funding for day programs, and applying for Medicaid waivers to cover in-home care.

The director of the complex care clinic described the case-manager/care coordinator role as indispensable. She connects patients and families to resources in the community and helps them navigate the many disparate pieces required for successful transition to adulthood. However, funding the case-manager role has been challenging. Because the position is funded through the organization’s health plan, the case manager only supports individuals with the health plan. Care-coordination for patients with other insurances falls on the physicians.

### Findings on RQ2 from semi-structured interviews

To address RQ2, we analyzed interviews with nine national transition experts guided by the RE-AIM implementation science framework. Demographics are in Table 1. Of the nine transition experts interviewed, three worked at a Geisinger transition clinic and six worked at transition clinics from health systems around the United States. Most interviewees were physicians (8/9) working in primary care (7/8), including pediatrics (4/8) and internal medicine (2/8). Qualitative findings from interviews are organized below according to RE-AIM construct (Tables 2, 3, 4, 5 and 6). Definitions of RE-AIM constructs are based on the analysis by Holtrop et al. [42], which describes qualitative dimensions of each RE-AIM construct (See esp. Table 2 w/in Holtrop et al. 2018).

**Table 1:** Demographics: expert interviews

Participants = 9			
Sex	Female, *n* = 5 (56%)	Male, *n* = 4 (45%)	
Age Ranges	35 – 44, *n* = 7 (78%)	55 – 64 (11%)	> 65 (11%)
Race & Ethnicity	Hispanic, *n* = 1 (11%) Arabic, *n* = 1 (11%)	Non-Hispanic, *n* = 8 (89%) White, *n *= 8 (89%)	
Role	Physician, *n* = 8 (89%)	Respiratory therapist/Program coordinator, *n* = 1 (11%)	
Specialty	Pediatrics, *n *= 4 (44%) Internal medicine, *n *= 3 (33%)	Pediatric pulmonology, *n* = 1 (11%)	Oncology, *n* = 1
Years in Practice	5–10, *n* = 3 (33%)	11 – 15, *n* = 4 (45%)	> 20, *n* = 1 (11%)
Years at transition clinic	5–10, *n* = 5 (56%) N/A, *n *= 2 (23%)	11–15, *n* = 1 (11%)	16–20, *n* = 1 (11%)
Healthcare organization	Baylor College of Medicine, *n* = 1 Children's Hospital of Philadelphia, *n* = 1 Geisinger, *n* = 3	Indiana University, *n* = 1 Nationwide Children’s Hospital, *n* = 1	Ohio State University Wexner Medical Center, *n* = 1 University of Chicago Medicine, *n* = 1

**Table 2:** Clinic observations: clinicians and patients observed

Clinic	Cystic Fibrosis Clinic	Complex Care Clinic
Clinicians observed	*n* = 9	*n* = 6
Clinician Type/Specialty	Pulmonologist, attending (*n* = 3); Nurse (*n* = 1); Respiratory therapist (*n* = 1); Genetic counselor (*n* = 1); Psychologist (*n* = 1); Dietician (*n* = 1) Social worker (*n* = 1)	Internal medicine physician, attending (*n* = 2) Internal medicine physician, resident (*n* = 2 Case manager (*n* = 2)
Patients observed	*n* = 5	*n* = 11
Condition	CF (*n* = 5)	EDS (*n* = 1); Epilepsy (*n *= 1); RS (*n* = 1); CP (*n *= 2) BS (*n* = 1*); KBG (*n* = 1); SB (*n* = 1); DD (*n* = 2); NF (*n* = 1); MD (*n* = 1)
Sex/Gender	Female (*n* = 1) Male (*n* = 4)	Female (*n* = 8) Male (*n* = 2) Transgender (*n* = 1)
Age	≤ 10 (*n* = 1), 11–14 (*n* = 1), 15–18 (*n* = 3)	19–22 (*n* = 7), 23–26 (*n* = 1), 27–30 (*n* = 3)

*BS* Blau syndrome

*CF* cystic fibrosis

*CP* cerebral palsy

*EDS* Ehler Danlos Syndrome

*DD* developmental delays, not otherwise specified

*KBG* syndrome caused by ANKRD11 gene

*MD* muscular dystrophy

*NF1* neurofibromatosis type 1

*RS* Rett syndrome

*SB* spina bifida

^*^Patient counted twice: also has CP

**Table 3:** Reach: key findings with exemplar quotations

Domain/Theme	Summary of findings	Exemplar Quotations
Initiating patient participation: referrals and clinic awareness	• Patients learned about the clinic through word of mouth from other patients and providers.	• *The biggest way that we've been able to continue to have patients come in is word of mouth. So, a lot of these families talk on social media. A lot of these families share nursing agencies or county resources, and they all talk… or they see their primary care doc and say hey, we heard about this clinic, I want a referral. (Clinician 1)*
Ongoing patient participation: follow-ups	• Patients were encouraged to return for follow-up appointments through reminders (manual and automated) and by anticipating and addressing barriers (psychological and logistical) to attendance.	*• [T]rying to convince people to go away from this once-a-year model to hey come in every 3 or 4 months so we can really start to chip away at some of these things, that can be kind of a tough sell, and we try to sell that and ensure people understand that's the expectation before they even walk in the door. (Clinician 8)*

**Table 4:** Effectiveness: key findings with exemplar quotes

Domain/Theme	Summary of findings	Exemplar Quotations
Measuring success	• Participants described metrics tracked to demonstrate clinic outcomes and successes. o Outcomes tracked included emergency room utilization, hospital admissions, length of hospital stay, increased preventative health screenings and vaccines, patient satisfaction, number of consults	• *The things we tracked were how many consults we were getting, what we did for the consults, the positive impact… For example, my consult service reduced the length of stay from eight days to seven days. That's an entire day saved. Which, over the course of like thousands of admission days, is actually rather large. (Clinician 9)*
Key elements for success and descriptions of successes	• Successes included putting patients at ease, moving patients from pediatric to adult care, supporting external clinicians who take on complex patients, and patient and provider satisfaction.	• *And we're successful at helping families alter their vision for what to do next. We spend a fair amount of time helping families un-bubble wrap children who are too heavily bubble wrapped to have a chance of being self-managing. (Clinician 7)* • *We actually found that providers who are referred to us were more comfortable discussing transition with other patients that they didn't refer to us, which was kind of neat and then again, that kind of marker with providers perceiving that we saved really towers of work for them. (Clinician 6)*

**Table 5:** Adoption: key findings with exemplar quotes

Domain/Theme	Summary of findings	Exemplar Quotations
Making the case to the institution (value: economic)	• Interviewees tracked metrics to prove the economic value of the clinic.	• *I think the first year of running the service and taking care of patients, we dropped the number of admissions by like 10% and since the creation of the service…I think I've dropped admission rates by 35 to 40%, which is a considerable number. I think…the important arguments I initially tried to make were based on cost savings to the medical system and showing the work. (Clinician 9)*
Making the case to the institution (value: organizational mission and goals)	• Interviewees discussed showing consistency with the organization’s values and practical priorities to justify a transition clinic.	• *Our county health system very much values serving underserved patients. So we serve highly disabled people with high levels of disability, both social and medical, and that is good for their mission of serving the vulnerable population. (Clinician 7)*

**Table 6:** Implementation: key findings with exemplar quotes

Domain/Theme	Summary of findings	Exemplar Quotations
**Funding and Clinic Structure**		
Funding and cost considerations	• Participants explained that clinics are unlikely to make money for an institution.	• *[I]f you're going to do this right, I think with the current billing the way it is, you're not going to end up, I don't see how you could possibly end up positive. I would love to hear if someone is able to do that, but the way things are, I don't see that as a real possibility. (Clinician 8)*
Funding sources and changes over time	• Clinics were funded by a combination of grants (internal and external), private donors, and home institution/organization, partnering institution.	• *Our transition clinic was initially grant funded…, so we did that for 2 years. It was kind of just proof of concepts or pilot program… then the program became kind of funded within operations at the Children's Hospital. (Clinician 6)*
Clinic structure and design	• The structure/model/home of transition clinics varied according to patient population, institutional needs, and organizational support.	• *[S]o my clinic is a Med-Peds Clinic, and within it…those of us that take care of this group of patients just build them into part of our primary care panel; and so they are coming into the same check in, mostly same staff. (Clinician 5)*
**Staff and Clinician Roles**		
Staff and clinicians-all roles	• Clinic staff varied depending on clinic structure, but generally included physicians, social workers, and nurse navigators or care coordinators.	• *[D]epending on how many team members need to see them or what their concerns are for the day…does a physician need to spend more time with them going over meds or do they need to see other team members for other reasons, whether it be the social worker or the dietician, or the genetic counselor, or the respiratory therapist. ( Clinician 3)‬‬‬‬‬‬*
Staff and clinicians: care coordination role	• Care coordinators were described as essential to the functioning of the clinic. • Interviewees emphasized the importance of going beyond medical needs to address social needs and needs between medical appointments.	• *[T]he clinic would not run without [case manager’s name], like I'll be completely honest. (Clinician 1)* • *[W]e used a combined nurse and social work model, and that is very useful for just dividing and conquering the social versus medical complexity needs. Social workers are better at the DPS issues and collaborating with the lawyers …whereas the nurses are really good at getting us durable medical equipment and home modifications… and health education…. So we…strongly believe in a trans-disciplinary model using nursing and social work.‬ (Clinician 7)‬‬‬‬‬*

#### RE-AIM domains

##### Reach

Interviewees described how they reached patients eligible to participate in the transition clinic (Table 3). Word of mouth from patients, families, and clinicians played an important role in increasing referrals. A few interviewees described having more patients than they could accommodate. Consequently, advertising the clinic was not a priority, but defining their target population with specific inclusion/exclusion criteria was essential for appropriate referrals. To improve ongoing participation in the clinic, some clinics used texting and phone call reminders. One interviewee described dividing current patients based on levels of self-advocacy to prioritize reaching the patients most in need. Education, of both patients and providers, was described as essential for setting expectations about appointment frequency and the level of commitment required for participation in the clinic.

##### Effectiveness

Interviewees explained which metrics they tracked, how they were tracked, and which outcomes were most meaningful for capturing clinic successes (Table 4). Most interviewees tracked and reported high levels of patient and family satisfaction. One interviewee explained that they track patient satisfaction separately from their institution to learn about dimensions of care that might otherwise be missed, such as emotional connection with a new doctor. Other interviewees described outcomes they found meaningful, but which might not be of interest to their institution. For example, interviewees described feeling proud of their clinic’s success in reducing patient anxiety, reducing workload for referring clinicians, and offering excellent training to residents and medical students.

##### Adoption

Interviewees described making the case for a transition clinic to their organization, including metrics used and justifications for adopting the proposed clinic (Table 5). Because transition clinics were described as unlikely to generate hospital revenue, interviewees stressed the importance of finding alternative ways to demonstrate clinic value. They described two aspects of clinic value that motivated adoption: economic value and consistency with organizational mission.

To demonstrate the economic value of the clinic, interviewees showed cost savings by pointing to lower emergency room utilization, fewer in-patient admissions, and shorter hospital stays. Several interviewees explained how their clinic increases appointment availability for non-transitioning patients. For instance, the clinic decreases the number of adults seen in pediatrics, which opens appointments for new pediatric patients; and the clinic reduces subspecialty referrals by meeting patient needs within primary care, which improves access to subspecialists for other patients.

Interviewees said that showing compatibility with the hospital’s mission and priorities was another dimension to demonstrating clinic value. For example, one interviewee emphasized the ways their transition clinic serves a vulnerable patient population because their health system values caring for underserved patients. While non-economic arguments were not sufficient for institutional adoption, participants described how they helped.

##### Implementation

Interviewees discussed the logistics of clinic funding and cost considerations, factors influencing structure and design of the clinic, and clinician roles (Table 6). They explained that providing good transition care is expensive because it requires long appointments and care coordination between appointments. Accordingly, they cannot turn over the same volume of patients as other practices.

Clinic models varied by institution. Some programs were large, multidisciplinary clinics, while others used a consult model with one or very few individuals. Clinician time committed to the program also varied from a few hours per week to full-time roles. Typically, interviewees said programs included some or all of the following clinicians: physicians, nurses, advanced care providers, social workers, and care coordinators.

Most interviewees emphasized the essential role of care coordination by social workers and nurses. They explained that much of the work required to support transition occurs outside of the actual appointment. Interviewees discussed how care coordinators and social workers assisted with intake calls, symptom assessment between appointments, planning for upcoming challenges with patients, coordinating follow-up imaging and labs, vocational counseling, waivers, insurance transitions, and accessing state-based services. One interviewee stressed the importance of having both a care coordinator and a social worker to address social and medical needs between appointments.

##### Maintenance

Interviewees described economic and non-economic factors that contribute to clinic maintenance (Table 7). A few interviewees explained how their programs used grant funding as proof of concept, but ultimately transitioned to institutional support by showing the value of the clinic. One interviewee said their program was funded in part by the state and the state has sustained this support over time. Other interviewees discussed how their programs relied on partnerships with other organizations or departments for sustainability.

**Table 7:** Maintenance: key findings with exemplar quotes

Domain/Theme	Summary of findings	Exemplar Quotations
Funding and financial support over time	• Participants described funding changes over time after the initial start-up phase of the clinic.	• *I guess maybe grants are necessary in the very beginning to prove that this is financially viable, so the institution doesn't have skin in the game. But my opinion is that the grants should only be for like very small pilot studies to get the data necessary to prove to the institution that it is viable. (Clinician 9)*
Continuing recognition of clinic value by organization and leadership	• Participants emphasized the importance of maintaining connections with leadership to ensure that belief in the value of the clinic continues.	• *I kind of started out with a lot of leadership support, a high level of administrative leadership support, but over the ensuing years as some of those leaders took different jobs… and no one replaced their position when they left, and so it left this void. (Clinician 2)*
Infrastructure and funding for sustainability	• To make their clinic sustainable, many clinics were housed within larger departments or partnered with departments with a stake in their success.	• *We do have a defined partnership with the pediatric side. They also help to some degree finance our clinic because with the additional support we have for care coordination, extended visit times, the other stuff that we have to really do this well, we’re not able to just meet the bottom line based on our billing alone. (Clinician 8)*
Non-economic reasons for sustainability (maybe organizational values, mission, priorities)	• Participants described reasons for the sustainability of the clinic that went beyond economics. • Examples included contributing to physician training, working within a population health model rather than an RVU model, and being an important part of the community.	• *[I]t's embedded in a neighborhood in one of the not greatest neighborhoods in [city], and so supports the hospital's larger mission of being committed to communities, particularly communities of need. And some of it is the philosophy of [healthcare system] where primary care is seen as the foundation from which the rest of the hospital sort of functions. And so if primary care is not maximizing profit or whatever, like, we'll figure that out elsewhere as an institution, right? (Clinician 4)*

Participants also described how clinic design and institutional structure played a role in making the program financially sustainable. For instance, one interviewee said the program exists within a system that uses a value-based, population-health model rather than a relative value units (RVU) model. This enables them to offer longer appointment times and focus on follow-up, care coordination, and quality improvement without the stress of maximizing RVUs. Other interviewees explained how their programs achieved similar sustainability by housing the clinic within a larger department to which they contributed by seeing non-transition-aged patients.

Interviewees also described non-economic reasons that contributed to their clinic’s sustainability. For example, one participant said their clinic is an important part of the neighborhood and serves the hospital’s larger mission of helping communities in need. Another interviewee described how their clinic plays an important role in resident education. A few interviewees said that continued recognition by and communication with leadership was also important for sustaining the clinic.

#### Barriers to transition care

Interviewees described barriers to providing transition care and maintaining a successful clinic (Table 8). They emphasized insurance as one of the primary barriers to care. In addition to patient challenges navigating the transition to adult insurance, providers experienced challenges to providing equitable care for patients. For instance, two interviewees said it was difficult to connect patients to specialty care because many subspecialists were unwilling to take Medicaid. Another interviewee explained that because there was no funding for an insurance-agnostic case manager/care coordinator, gaps in care-coordination for patients with certain insurances fell to physicians. Several interviewees described the impact on transition care when their state expanded or did not expand Medicaid coverage.

**Table 8:** Barriers key findings with exemplar quotes

Domain/Theme	Summary of findings	Exemplar Quotations
Reaching patients/clinic attendance	• Participants described barriers to patient attendance, such as distance to clinic and access to transportation.	• *Distance is a huge thing because we see patients from all over the Northeast…some of them are driving two or three hours to get here for the clinic visits, and then they get tired, and that's another thing, the clinics are long and sometimes the older kids are like I've just had enough and they don't want to, if we start talking transition and asking questions, they want no part of it. (Clinician 3)*
Insurance	• Participants described challenges related to insurance. • Examples included providers unwilling to accept Medicaid and insurance companies not covering care.	• *I think the hardest part [is] we don't necessarily have access to all the pieces that you could possibly want to best support this population….[I]t's hard to find people who take Medicaid particularly within some of the subspecialties. (Clinician 8)*
Staff turnover and training	• Finding, training, and keeping staff was a challenge that impacted care provided.	• *Haven't talked about hiring new people and training them. And it is one of the biggest challenges…[H]iring people who understand that problem-solving for people with disabilities and complexity requires getting outside the box as often as the rules fit and needs people who have enough creativity and self-direction to adequately achieve outcomes. (Clinician 7)*
Workflow challenges	• Participants described challenges related to workflow Processes that were usually automated in other contexts were manual for them.	• *We're constantly needing to re-educate our scheduling people which has been a challenge, and if they don't reschedule them right, then they may not get tracked…so then we're having to go back and change things. So, tracking and scheduling is hard, because it’s a special population within an existing system. (Clinician 5)*

Participants also identified barriers related to workflow. Many processes automated in other contexts were manual in transition clinics. For instance, one interviewee said she was on her third social worker in five years, not because they do not like the program, but because the pediatric hospital system has age limits that undermine the social workers’ ability to care for adults. This affected other specialists in the program too because they had trouble placing orders for adults in an EMR built for children. Another interviewee described how clinicians created processes on a case-by-case basis whenever patients needed inpatient care or imaging because their clinic is outside of the hospital.

## Discussion

Health care transition for AYA with hereditary cancer predisposition can prepare young adults to continue cancer screening and other cancer prevention throughout adulthood, thereby increasing early detection of cancers and decreasing morbidity and mortality from preventable cancers. To inform design of a future transition clinic for AYA with hereditary cancer predisposition, we conducted a contextual inquiry of transition clinics at Geisinger and interviews with national transition experts. We combined HCD and implementation science to identify key elements of successful clinics at Geisinger as well as barriers and facilitators relevant to designing a new clinic for AYA with hereditary cancer predisposition. Pairing HCD and implementation science enabled us to center clinician experiences during pre-implementation planning and specify what works for the people who provide transition care.

### Contextual inquiry

The two clinics observed used different models of care to support their unique patient populations. However, certain key elements across both models contributed to their success and could be relevant to developing a transition clinic for AYA with hereditary cancer predisposition. Figure 3 shows overlapping clinic elements: sustainable clinic home, relationship building with patients, stepwise transition education for patients and parents, care coordination, and provider communication. These elements are consistent with recommendations by the Clinical Report on Health Care Transition and the organization Got Transition^®^ [65].

Our findings indicate that different models can be successful at the same institution. Many models of transition are reported in the literature, and there is no evidence that one is superior to another [20, 28, 52]. Studies frequently recommend choosing the model that best fits the practice and healthcare setting [48, 51, 52]. Additional research on patient and clinician preferences, as well as system-level priorities and goals, may clarify which model is ideal for AYA with hereditary cancer predisposition at our institution.

In both clinics observed, whole-person care was facilitated through relationship building between the clinician and patient, and a clinic for AYA with hereditary cancer predisposition will also need to prioritize relationship building. Relationship building was achieved in several ways: frequent appointments (every 3 months for both clinics), care-coordination, and education about social and medical dimensions of transition. Addressing the social dimension of transition was achieved differently in each clinic.

Clinicians at both clinics emphasized care coordination as integral to the success of their clinics. Not surprisingly the essential role of care coordination is also emphasized by the literature on transition care for AYA at risk for cancer [5, 48, 51, 52]. Regardless of condition, AYA appear to benefit from support around insurance, appointment reminders, connection to state resources, transportation, and other between-appointment needs.

The results of our contextual inquiry also indicate the importance of attention to clinician experience when designing a clinic that incorporates relationship building, patient education, and care coordination. Sufficient time for relationship building and funding for an insurance agnostic care coordinator are needed for the success of these three aspects of care. A systematic approach to patient education (e.g., using a checklist integrated into the electronic medical record system) ensures AYA are supported through each stage of transition by facilitating communication between the multidisciplinary team members who are responsible for different aspects of patient education. How a transition clinic for AYA with hereditary cancer integrates these dimensions into its design will depend on contextual factors, such as available staff and clinicians, clinic home and structure, and institutional resources and priorities.

### Interviews with transition experts

The implementation considerations described by national experts practicing in transition programs around the country overlap with and complement the clinic features observed in our contextual inquiry. Models of care varied from institution to institution; interviewees acknowledged that there is no one right way to design and implement a program, but care coordination was essential. Applying the RE-AIM theoretical framework to their feedback on development, implementation, and maintenance of transition programs identified important considerations for creating a transition program for AYAs with a hereditary cancer predisposition. As emphasized above, attention to clinician experiences uncovered nuances relevant for developing a new program.

In general, effectiveness outcomes described by interviewees were consistent with the literature on HCT for AYA with special healthcare needs that use the Triple Aim (and now Quintuple Aim) measures recommended by Berwick and colleagues to improve the U.S. health care system: patient experience, population health, cost of care/utilization, clinician experience, and equity [10, 13, 28, 49, 53, 59]. However, our results emphasize the importance of asking relevant stakeholders which measures they find meaningful, as goals and definitions of success will be different for clinicians, administrators, and patients [42]. For example, interviewees described, and sometimes tracked, successes that they, as clinicians, considered meaningful, e.g., patient connection to a new physician, even if not important for demonstrating clinic effectiveness to their institution.

Clinic value and costs were central to interviewee discussions about program adoption, implementation, and maintenance and will likewise be essential for planning a clinic for AYA with hereditary cancer predisposition. Others have also described the challenge of maintaining a transition clinic, particularly in view of the time investment required and poor reimbursement rates for essential aspects of care (e.g., care coordination) [19, 48, 63]. Less frequently discussed in the literature is making the case for institutional adoption by showing value in other ways, for instance, contributing to the hospital’s mission. Interviewees described knowing in advance that their program would not be profitable for their institution. Consequently, they focused on a more expansive conception of value, e.g., improving resident education or fulfilling a hospital’s mission to contribute to the local community and neighborhood. This helped them address barriers related cost-effectiveness.

Interviewees described insurance and the manual nature of clinical workflow as barriers to implementing and sustaining coordinated, whole-person care. While insurance is frequently discussed in HCT literature, it is often from the perspective of patients, not clinicians [4, 26, 36]. However, clinician experiences may inform program design and improve fidelity to an intervention by anticipating roadblocks, such as poor Medicaid acceptance among subspecialists. Similarly, clinic design can be informed by clinician experiences with workflow, particularly challenges around manual processes that could be automated.

One of the greatest costs to implementing and maintaining a transition program is time (e.g., long appointments, care coordination). Addressing barriers in advance could improve efficiency in some areas and decrease pressure to curtail time-intensive dimensions of transition care. Improving workflow efficiency could also alleviate redundancy in staff roles and improve adoption as well as retention among clinicians and clinical staff.

### Limitations

This study’s contributions should be interpreted within the context of several limitations. First, data collection for both methods was limited to transition programs at six large healthcare organizations, including Geisinger, with established transition programs. It may have been fruitful to interview clinicians trying to establish new programs and clinicians who were unable to maintain their programs over time. Second, none of the individuals observed or interviewed cared for individuals with hereditary cancer predisposition. Because the clinicians interviewed practice in different geographical regions, in different specialties, using different models of care, some of their perspectives may not be relevant to implementing a clinic for AYA with hereditary cancer. Services needed by currently healthy AYA with hereditary cancer predisposition are different than those needed for individuals with chronic conditions. For instance, AYA with hereditary cancer will need increased surveillance to detect cancers and they may need psychological support for previvor status in the context of family losses to cancer. However, the feedback from clinicians in other specialties was salient to transition programs broadly. They provided key insights for implementation planning, as well as a strong foundation for creating a protocol for an AYA hereditary cancer predisposition transition program.

### Future research

To complement this study’s focus on clinician perspectives, more research is needed to understand the needs of patients and families with hereditary cancer predisposition. Learning about the goals, challenges, and priorities of this population may clarify which transition model(s) would best serve their needs. The clinicians we interviewed also identified a key area of future exploration: how healthcare systems value and support HCT programs. Understanding the perspective of payers and systems is essential to implementing programs sustainably. PRISM, an implementation science framework that includes RE-AIM plus four contextual domains, could help structure future studies exploring multi-level contextual factors, including system-level perspectives, that influence implementation outcomes [35].

Future research can also consider how new technologies could facilitate transition and improve the workflow challenges identified as barriers by our study participants. Clinician- and patient-facing applications could play an important role in educating patients and clinicians about condition-specific recommendations across the lifespan to reduce cancer risk for individuals with hereditary cancer predisposition.

## Conclusion

We used HCD and implementation science to inform development of an HCT program to be piloted in the hereditary cancer space. Learning from clinicians who have designed, implemented, and operated a transition clinic can inform implementation planning by centering people and their context to ensure that the correct problems are identified prior to designing innovative solutions. We identified clinician perspectives on factors specific to reaching the target population, organizational adoption, tracking effectiveness, and maintenance. Our results affirm the findings of other studies that relationship building, patient education, and care coordination are essential elements to HCT. While we found that multiple models of HCT can work within one healthcare system, patient needs, provider availability, and system-level factors will dictate which models are most effective and sustainable for our population of interest. The results of this study will inform the next phase of this project to plan an HCT program for AYA with hereditary cancer predisposition.

## Data Availability

No datasets were generated or analysed during the current study.
